# tDCS effects in basic symbolic number magnitude processing are not significantly lateralized

**DOI:** 10.1038/s41598-023-48189-z

**Published:** 2023-12-06

**Authors:** Narjes Bahreini, Christina Artemenko, Christian Plewnia, Hans-Christoph Nuerk

**Affiliations:** 1 Department of Psychology, University of Tuebingen, Tuebingen, Germany; 2 Department of Psychiatry and Psychotherapy, Neurophysiology and Interventional Neuropsychiatry, University Hospital of Tuebingen, Tuebingen, Germany; 3 German Centre for Mental Health (DZPG), Jena, Germany

**Keywords:** Cognitive neuroscience, Psychology

## Abstract

Functional lateralization was previously established for various cognitive domains—but not for number processing. Although numbers are considered to be bilaterally represented in the intraparietal sulcus (IPS), there are some indications of different functional roles of the left vs. right IPS in processing number pairs with small vs. large distance, respectively. This raises the question whether number size plays a distinct role in the lateralization within the IPS. In our preregistered study, we applied anodal transcranial direct current stimulation (tDCS) over the left vs. right IPS to investigate the effect of stimulation as compared to sham on small vs. large distance, in both single-digit and two-digit number comparison. We expected that anodal tDCS over the left IPS facilitates number comparison with small distance, while anodal tDCS over the right IPS facilitates number comparison with large distance. Results indicated no effect of stimulation; however, exploratory analyses revealed that tDCS over the right IPS slowed down single-digit number processing after controlling for the training effect. In conclusion, number magnitude processing might be bilaterally represented in the IPS, however, our exploratory analyses emphasise the need for further investigation on functional lateralization of number processing.

## Introduction

Magnitude understanding has been a necessary cognitive ability, which can be found in animals for human beings outside modern civilizations for non-symbolic stimuli^1–6^. To survive, our ancestors and other animals had to make sure their food supply is holding out well while correctly counting the number of attackers to their realm. Now, living in the modern era humans still encounter numbers for our most basic needs, but in a more formal symbolic form, for example, when awaiting for a flight departure or tracking the time in a meeting. From our ancestors to us, we seem to be equipped with two core systems for numerical representations^7^. To study the innate nature of this systems they have been tracked into human infancy. For example, abstract numerical representation^8^ and large-number discrimination^9^ has been shown in infancy and a series of experiments were accomplished to distinguish object-based and enumeration-based representation^10^.

A basic everyday numerical skill which serves as an indicator of magnitude understanding is the ability to compare numbers relative to their magnitude size. Comparison between two numbers leads to a phenomenon so-called numerical distance effect (NDE). According to the distance effect, comparing numbers is faster and more accurate when the numerical distance between two numbers is relatively larger (e.g., 2 vs. 9), than when it is relatively smaller (e.g., 8 vs. 6)^11^. The NDE is assumed to arise from a noisy mapping between numbers located on a mental number line which is commonly used to characterize magnitude representation in a wide range of subjects from adults^12,13^, preschool children^14^ to new-borns^15,16^, and even has been tracked in animal studies^17–19^. The so-called spatial-numerical association of response codes (SNARC) effect is a classic example of this relatedness between numbers and spaces. It explains that during magnitude comparison left-side vs. right-side behavioral decisions are faster for relatively small vs. large numbers, and vice versa^20^. According to the most dominant model of the SNARC effect, numbers are represented on a spatial line from the left side to the right representing small to large magnitudes (at least in Western societies)^21^. However, it has been argued that the SNARC effect is flexible and relies on relative magnitudes (smaller/left vs. larger/right)^22,23^ rather than absolute ones (small/left vs. large/right), it can be influenced by symbolic vs. non-symbolic magnitudes differently^24^ and it can be modulated by cardinality feature of numbers, working memory^25^ or a multiple coding account^12^. We will get back to some of these distinctions about (spatial) magnitude processing later.

Data from functional neuroimaging studies as well as brain-damaged patients have revealed that regions in and around the intraparietal sulcus (IPS) are involved in magnitude processing and hence influenced by numerical distance. Activation within the IPS is negatively correlated with numerical distance in magnitude comparison tasks^26–28^. This pattern of activation has been reported by fMRI studies on healthy samples including adults and children^27,29–33^.

The Triple Code Model of numerical cognition and its successors^34,35^ has also argued for the involvement of bilateral IPS in magnitude processing. This model suggests distinct but overlapping neurocognitive mechanisms underlies the three primary representational domains of number and bilateral IPS is the main and common brain region involved in all representational codes^33^. In addition, in patient studies the IPS has been found to be essential^36^.

Hence, the IPS is a core region for the representation and processing of magnitude information (for a meta-analysis see^37^). Nevertheless, the IPS is not the only region involved in magnitude processing but embedded in a fronto-parietal network of number processing^38–42^. In addition to parietal brain regions such as the IPS, frontal regions such as the inferior frontal gyrus, Brodmann area 47, the supplementary motor area together with their connections are involved in number processing^43^. Now the question is if magnitude processing is bilaterally represented in the IPS within the fronto-parietal network or if magnitude processing is lateralized to the right or left IPS dependent on specific magnitude features. Interestingly, the functional lateralization of magnitude processing in the IPS has not yet been as systematically studied as it has been for other cognitive functions like language or attention.

Evidence for lateralized activation in the IPS during magnitude processing can be categorized based on different features of the magnitude information, namely, (1) the format in which numbers are represented (symbolic vs. non-symbolic), (2) the automatic or effortful way of magnitude processing, and (3) the size of the magnitude (small vs. large).

First, the human’s ability to represent numbers either symbolically such as (2) or (two), or non-symbolically (••) has been recognized by Triple Code Model. Accordingly, empirical research has highlighted differential patterns of brain activation for numerical stimuli based on stimulus format^44–47^. For example, right lateralized parietal and frontal regions showed greater activation for non-symbolic compared to symbolic addition^48^ and left IPS has been shown to be more finely tuned to symbolic Arabic numbers compared to non-symbolic dots^47^.

Second, hints for lateralization were found for the level of cognitive demand needed in processing magnitudes. The left IPS seems responsible for effortful processing, whereas the right IPS is more involved in overlearned or automatized processing of numbers^49–52^. In addition, there is evidence that exact compared to approximate numerosity judgments are associated with greater activation of a left-lateralized fronto-parietal network^53^. Thus, a functional asymmetry of the IPS can be postulated along an effortful–automatic continuum of cognitive demand^28^.

Third, along with other influential factors, Pinel et al.^54^ in a fMRI study found that the left and right parietal cortices are more involved in comparing number pairs with a small and large distances, respectively. Supporting the fMRI data, non-invasive brain stimulation methods such as transcranial magnetic stimulation (TMS) and transcranial electric stimulation (tES) allow for investigating the causal involvement of IPS in magnitude processing. For instance, single (inhibitory) pulse TMS over the left and bilateral posterior parietal cortex including the IPS resulted in a deficit to compare single-digit numbers^55,56^. Interestingly, left stimulation had a stronger influence on close numbers and bilateral stimulation on numbers with larger distance. For instance, a patient with an infarct restricted to the left IPS showed deficits in basic magnitude processing tasks, including a larger distance effect compared to the matched control^57^.

Taken together, although The Triple Code Model suggested the bilateral involvement of IPS in magnitude processing, the literature also shows evidence for functional lateralization with more pronounced left-hemispheric activation in case of small distance. The functional involvement of the left and right IPS in magnitude processing—in terms of a causal structure-function relationship—cannot be inferred from neuroimaging data. The reason is that brain activation is the dependent variable in neuroimaging studies and thus activation may be necessary for performing the task but may also be mere co-activation (from adjacent structures, the homologue contralateral area, or the network) or dysfunctional activation (due to inefficient or compensatory resource allocation) without any functional relevance. However, brain stimulation methods can causally bolster the correlational evidence from neuroimaging and allow for investigating the functional involvement of cortical sites identified by fMRI in a given task (for a review see^58^). By stimulating a specific brain area, the neural activity of this target area can be influenced by shifting cortical excitability^59^, thus, brain activity becomes an independent, modifying variable rather than a dependent, measurable one.

Studying this causal relationship, Hauser et al.^60^ conducted a transcranial direct current stimulation (tDCS) study to investigate the role of the IPS on number magnitude comparison as well as two-digit subtraction. Anodal tDCS over the left IPS—but not cathodal tDCS or bilateral tDCS—increased accuracy in magnitude comparison and subtraction. In contrast, magnitude processing was facilitated after anodal tDCS and inhibited after cathodal tDCS over the bilateral IPS^61^ but not after unilateral stimulation^62^, supporting the assumption of a bilateral involvement of IPS. On the one hand, these results show the potential of tDCS to influence number magnitude processing, and on the other hand, point at the controversial findings on hemispheric laterality in this domain. The question remains whether the neural core representation of number processing is bilateral or lateralized. The idea is that the left IPS is more involved in small distance and effortful number processing and the right IPS in large distance and automatized processing—this causal relationship between the IPS and magnitude processing will be investigated in the current tDCS study.

Another important point in numerical cognition is that our knowledge of magnitudes has been dominated by research on single-digit numbers (1 to 9) and often implicitly generalized to multi-digit integer numbers at large. However, these two number ranges significantly differ from each other^63^. Contrary to single-digit numbers, multi-digit numbers have in addition to the overall magnitude a place-value structure which assigns a value to each digit depending on their position in the number (e.g., units, decades). Therefore, in two-digit numbers the overall magnitude is not processed as a whole, but in a decomposed manner^64–66^. The distance effect occurs in two-digit magnitude comparison tasks, too^67,68^, however, the unit-decade compatibility effect indexing place-value processing can also modulate responses. The compatibility describes that in a two-digit number comparison task reaction time is decreased and accuracy is increased when the unit and decade comparisons lead to the same decision (for example, 56 and 41 with 5 > 4 and 6 > 1 vs. 51 and 36 with 5 > 3 but 1 < 6)^63,64,68^. Overall distance is typically matched between compatible and incompatible number pairs; therefore, an overall analogue magnitude representation cannot explain this effect, but decomposed place-value processing can explain it (for a model see^69^). Thus, the compatibility effect supports the notion that two-digit differ from single-digit numbers. This is not merely an academic distinction without any relevance to applied matters. For instance, Ashkenazi and colleagues showed that dyscalculics differ from controls in distance effects for two-digit numbers, but not for highly overlearn single-digit numbers. Therefore, any study claiming to investigate number processing per se and not only the highly overlearn single-digit numbers, should go beyond the single-digit number range. This is what we will do here.

Although evidence from single-digit and two-digit magnitude processing has been mostly presented together, some behavioural studies started differentiating these number magnitudes—while neuroscientific studies are still very rare. Goebel et al.^70^ in a TMS study observed a dissociation of the neural correlates in magnitude comparison for single-digit and two-digit numbers: magnitude comparison of single-digit numbers was affected by TMS over the right anterior IPS, while two-digit numbers by TMS over the posterior bilateral IPS. This observation can be served as a starting point for paying attention to possible distinct neural underpinning of single- and two-digit numbers.

The current study focuses on the effect of magnitude size from the causal functional-structural perspective by means of tDCS in both single- and two-digit number magnitude comparison. Considering previous tDCS studies on magnitude processing which used anodal stimulation over the IPS and observed stimulation effects during two-digit number comparison task (mainly Hauser et al.^60^ who investigated brain stimulation on magnitude comparison), we followed the stimulation protocol and used unilateral anodal tDCS to causally investigate the functional lateralization of IPS during the symbolic magnitude comparison, considering both small and large distances. As preregistered (Study 3 in https://aspredicted.org/6B3_59S), we expected that anodal tDCS over the left IPS vs. sham facilitates magnitude comparison with small distances, while anodal tDCS over the right IPS vs. sham facilitates magnitude comparison with large distances. In this study, the effects of tDCS are measured separately for single-digit and two-digit numbers to investigate distance effects for different number magnitudes.

## Methods

### Participants

An a priori power analysis was conducted to determine sample size using the G*Power version 3^71^. Sample size estimation for the effect size of Cohen’s *d* = 0.2, with a power of 0.9 and *α* = 0.05 resulted in a minimum sample size of 53 participants. The power of 0.9 was chosen to maximize power in the current study. Cohen’s d of 0.2 indicates a small effect size and was used as typically found stimulation effects are relatively small (e.g., Hartmann et al.^72^ reported a significant effect of tDCS on subtraction ${\eta }_{p}^{2}\upeta{p}^{2}$ = 0.18 was reported) and this is the minimal effect size we were interested in.

The final sample consisted of 54 adults (18 males, age: *M* = 22.94 years, *SD* = 3.84 years, *Range* = 18–34 years). To keep the design counterbalance, at the end we recruited three more participant and replaced them. All participants were right-handed as assessed by the Edinburgh-Handedness Inventory^73^, non-smokers, native German speakers, with no history of neurological or psychiatric disorders. Furthermore, tDCS exclusion criteria have been adopted.

Participants were recruited using circular mails, private contacts and social media. As compensation, participants could either get course credits or receive monetary reimbursement. The local ethics committee for psychological research at the University of Tuebingen approved the study [Nuerk_2021_0902_235], and the experiment was performed in accordance with the relevant guidelines and regulations. Each participant gave written informed consent to participate in the study and to online access to the data.

### Material

**Figure 1:**
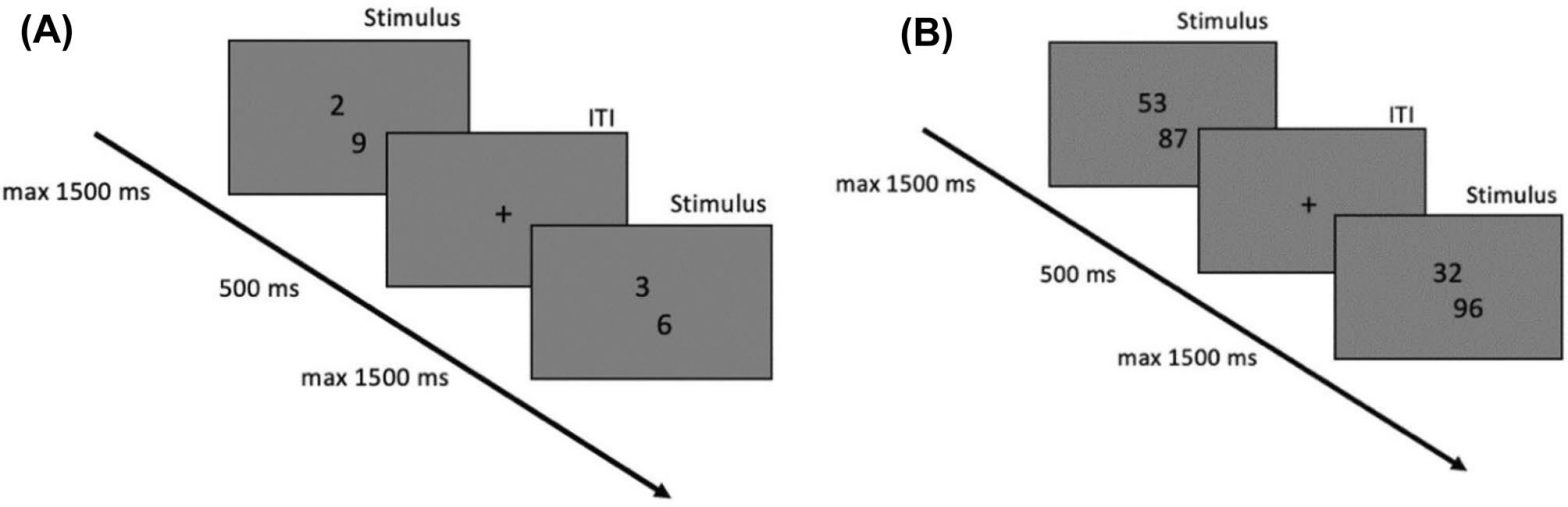
Number comparison task with (**A**) single-digit and (**B**) two-digit numbers.

In a symbolic number magnitude comparison task (see Fig. 1), pairs of two Arabic numbers were presented and the participants were asked to judge as fast as possible (each trial did not last longer than 1.5 s) which number is larger. The task was conducted in two versions: single-digit and two-digit. Single-digit number pairs consisted of 240 pairs of numbers between 1 and 9. Distance was manipulated to be either small (1–4) or large (5–7). Problem size and position of the larger number were counterbalanced across conditions. For two-digit number comparison, we used the data set of Nuerk et al.^64^ consisting of 240 between-decade pairs of numbers between 21 and 98. Distance was manipulated to be either small (11–39) or large (41–89), i.e., small (1–3) and large (4–8) decade distances. All number pairs consisted of four unique digits. Unit-decade compatibility, problem size and position of the larger number were counterbalanced across conditions.

The tasks were presented on a computer screen using PsychoPy^74^. The two numbers were displayed in font Arial size “24” in the centre of a 21″ screen in a vertical arrangement; the numbers were horizontally jittered by one digit. The arrangement of response keys on the keyboard was up and down; half of the participants were instructed to respond using the upper key with their dominant hand and the lower key with their non-dominant hand and vice versa. The response time limit was 1500 ms and a fixation cross was presented for 500 ms between the trials.

### Transcranial direct current stimulation (tDCS)

A DC-Stimulator MC (NeuroConn GmbH, Ilmenau, Germany) was used as constant direct current source. A current of 1 mA was applied on the head surface using rubber electrodes, covered with saline-soaked sponges. The active electrode (5 × 7 cm^2^) was placed over P3 or P4 of the international 10–20-system^75^ and the reference electrode (10 × 10 cm^2^, current density of 0.01 mA/cm^2^) over the contralateral supraorbital region—due to the large size of the reference electrode, this placement is expected to have minimum influence on the underlying brain region^59^. The arrangement of parietal cortex—contralateral supraorbital region was already successfully used in other tDCS studies^29,60,62,76^.

For active stimulation, the current was applied for 25 min and ramped up and down for 15 s. For left anodal tDCS, the target electrode was placed over P3 and a reference electrode over right supraorbital; for right anodal tDCS, the target electrode was placed over P4 and a reference electrode over left supraorbital. A bilateral electrode placement was used to blind participants with respect to the stimulation condition. In a bilateral placement, one channel follows the experimental protocol while the other channel follows the sham protocol in order to blind participants with respect to the stimulation condition. For sham, the current was applied only for 30 s after the 15 s of ramp up. This placebo-condition is known to be indistinguishable from active stimulation by the participants^60,77^. According to our bilateral placement, in the sham condition one channel followed the sham protocol (half of the participants left, the other half right) while the other channel was not delivering any electricity.

### Procedures

In a single-blind, within-subject design, each participant received left anodal tDCS, right anodal tDCS, and sham in 3 separate sessions with a minimum intersession interval of 4 days (*M* = 6.879, *SD* = 1.551, *Range* = 4–10 days). The order of stimulation conditions (left IPS, right IPS, and sham) were counterbalanced across participants. Each session followed the same procedure: 5 min after the onset of the respective tDCS protocol (25 min in total), participants conducted the two number comparison tasks. The order of the single-digit and two-digit number comparison tasks was counterbalanced across participants.

### Data preprocessing

Data analysis was conducted using R (Core Team, 2021) including ez^78^ and ImerTest^79^ packages separately for single-digit and two-digit magnitude comparisons according to our preregistration (Study 3 in https://aspredicted.org/zj7r8.pdf). No participant was excluded due to an accuracy rate lower than 75%. In the analysis of reaction times (RT), only correct trials and RTs between 200 and 1500 ms were included. Furthermore, RTs above and below 3 *SD* of the participant’s *M* were excluded from the data in a repetitive trimming procedure.

## Results

### Confirmatory analyses

As preregistered, repeated-measures ANOVAs and Bayesian ANOVAs with the within-subject factors stimulation (left IPS vs. right IPS vs. sham), distance (small vs. large) and their interaction were conducted on RT separately for single-digit and two-digit number comparison. Paired *t*-tests were further planned to compare tDCS of the left IPS vs. sham for small distances and tDCS of the right IPS vs. sham for large distances. Data are openly shared (https://osf.io/g57av/).

**Figure 2:**
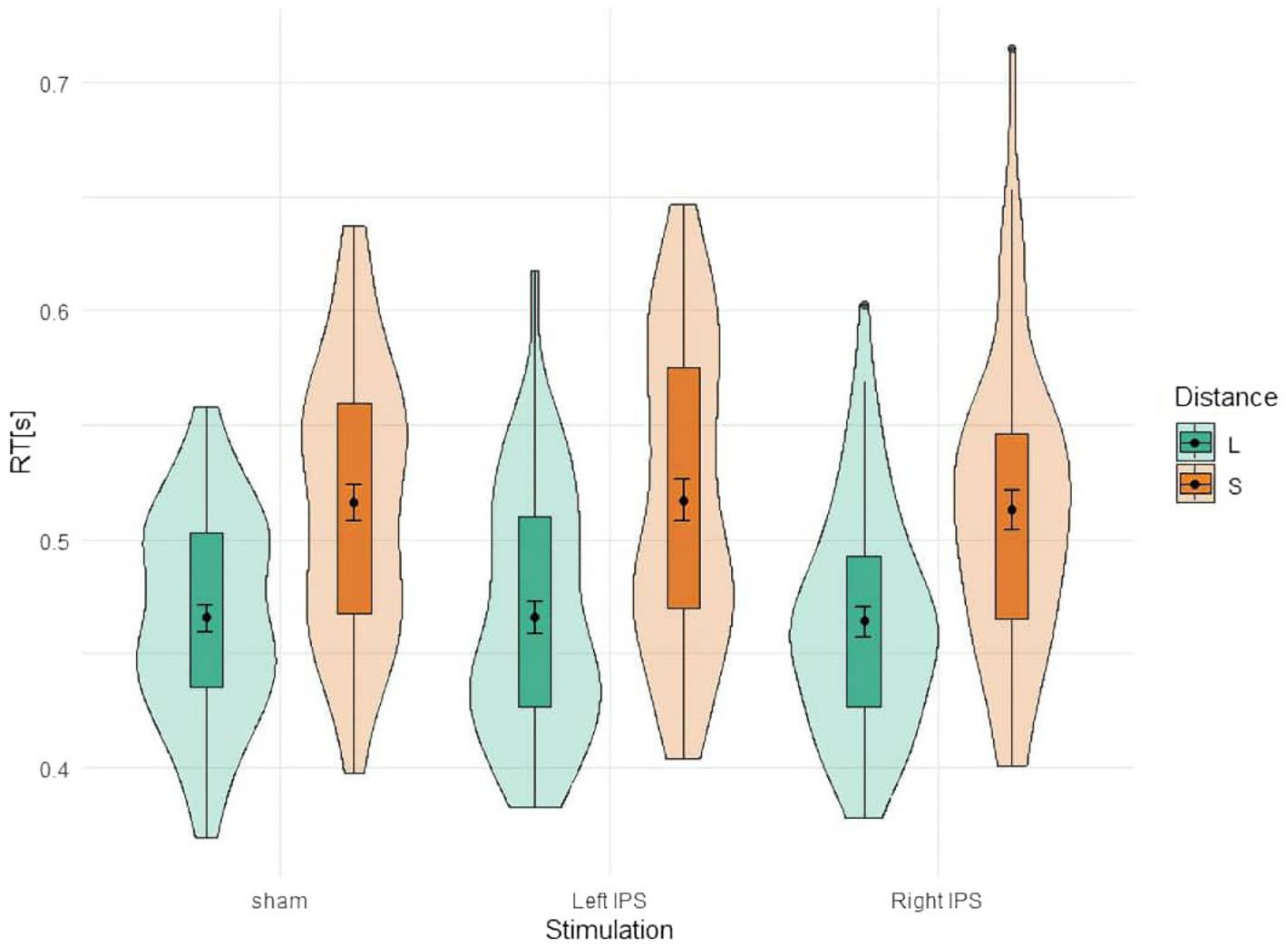
Mean reaction time after tDCS over the right and left IPS during single-digit number comparison. The green violins represent large distance and orange violins represent small distance. Error bars indicate standard deviation (*SD*).

The analysis of RT for single-digit magnitudes (overall *M* = 490, *SD* = 0.061 ms) showed a significant main effect for distance (*F*(1,53) = 324, *p* < 0.001; ${\eta }_{p}^{2}$ = 0.859), indicating that number comparison was significantly faster for large (*M* = 465, *SD* = 48 ms) as compared to small distances (*M* = 516, *SD* = 62 ms; see Fig. 2). There was no significant main effect of stimulation (*F*(2,106) = 0.18, *p* = 0.839, ${\eta }_{p}^{2}$ = 0.003), nor a significant interaction of distance and stimulation [*F*(2,106) = 0.42, *p* = 0.661, ${\eta }_{p}^{2}$ = 0.008]. We complemented the result by Bayesian analysis and there was evidence for a null effect for the interaction (*BF*_*excl*_ = 7.57). A BF between 1 and 3 indicates anecdotal evidence, a BF between 3 and 10 moderate evidence, a BF between 10 and 30 strong evidence in favour of one hypothesis^80^. The *t*-tests revealed no significant difference and even null effects for tDCS over the left IPS vs. sham for small distances (*t*(53) = 0.201, *p* = 0.841, *BF*_*01*_ = 6.61) nor for tDCS over the right IPS vs. sham for large distances (*t*(53) = − 0.367, *p* = 0.715, *BF*_*01*_ = 6.32).

**Figure 3:**
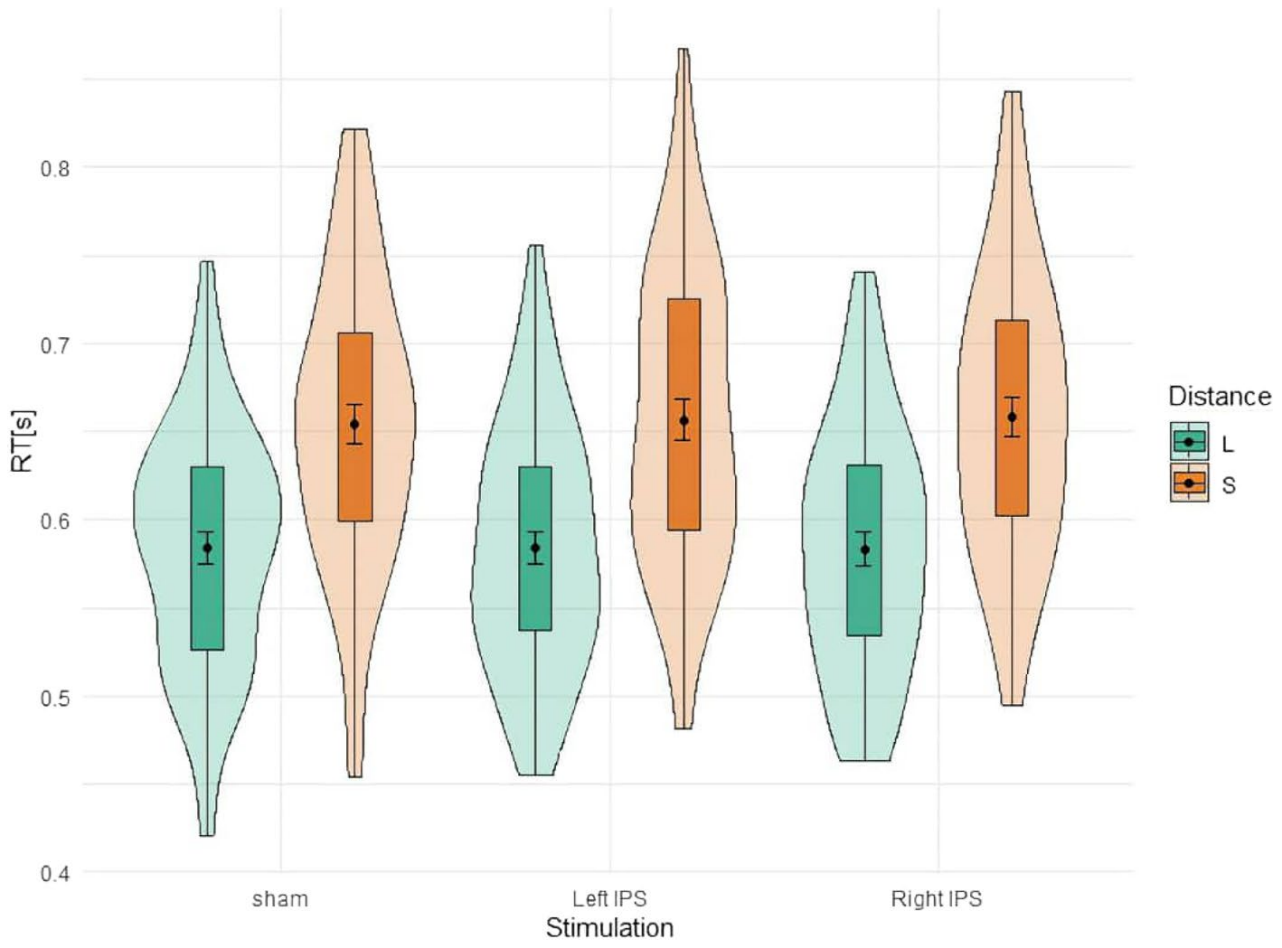
Mean reaction time after tDCS over the right and left IPS during two-digit number comparison. The green violins represent large distance and orange violins represent small distance. Error bars indicate standard deviation (*SD*).

The analysis of RT for two-digit magnitudes (overall *M* = 620, *SD* = 83 ms) showed a significant main effect for distance (*F*(1,53) = 508, *p* < 0.001, ${\eta }_{p}^{2}$ = 0.906), indicating that number comparison was significantly faster for large (*M* = 584, *SD* = 68 ms) as compared to small distances (*M* = 656, *SD* = 82 ms; see Fig. 3). There was no significant main effect of stimulation (*F*(2,106) = 0.043, *p* = 0.958, ${\eta }_{p}^{2}$ = 0.000), nor a significant interaction of distance and stimulation (*F*(2,106) = 0.781; *p* = 0.460; ${\eta }_{p}^{2}$ = 0.015). We found evidence for a null effect for the interaction (*BF*_*excl*_ = 81.67). The *t*-tests revealed no significant difference and even null effects for tDCS over the left IPS vs. sham for small distances (*t*(53) = 0.347, *p* = 0.730, *BF*_*01*_ = 6.36) nor for tDCS over the right IPS vs. sham for large distances (*t*(53) = − 0.029, *p* = 0.977, *BF*_*01*_ = 6.74).

### Exploratory analyses

**Figure 4:**
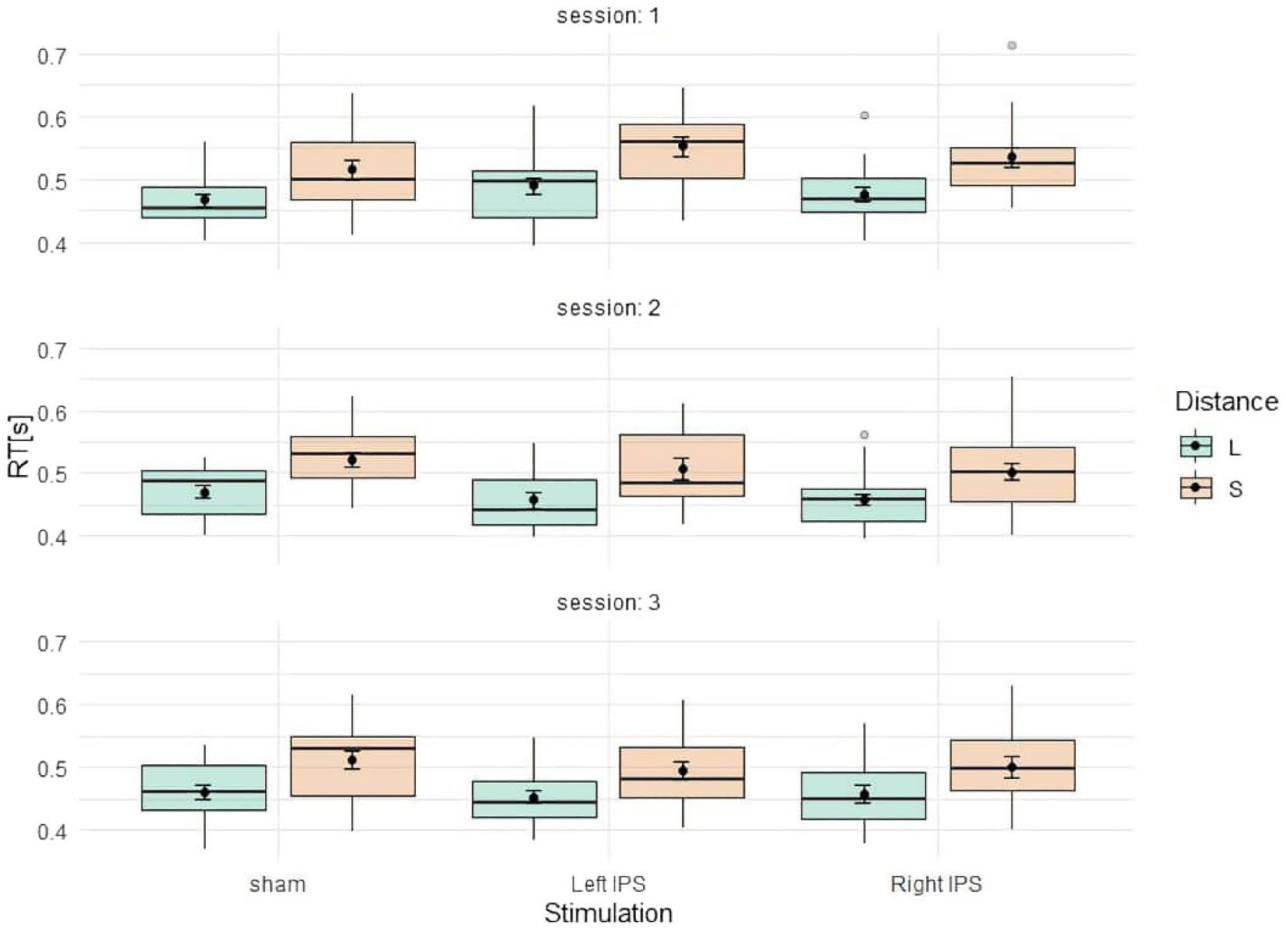
Influence of session on single-digit number comparison. Green represents large distance and orange represents small distance. Panels represent the sessions 1, 2 and 3. Error bars indicate standard deviation (SD).

This study was conducted in a within-subject design and thus each participant underwent three sessions with the same number comparison task. This led to training effects which might have overlayed possible stimulation effects (see Fig. 4). For exploratory analysis, we therefore reanalyzed the data by using a Linear Mixed Effects Model (LMM) on RT including the fixed factors stimulation (left IPS, right IPS, sham), distance (small, large), their interaction, and session (1, 2, 3) and participants as random factor. To obtain *p* values for the analyses of the effect of fixed factors we used the R package lmerTest, which calculates degrees of freedom using the Satterthwaite approximation. For model comparison, an automatic backward LMM selection procedure was applied which eliminates non-significant terms (*α* = 0.05 for both fixed and random effects) with the step function of lmerTest. The resulting reduced LMM for both single-digit and two-digit number comparison included significant effects for distance and session (see Supplementary Information: Table 1). Moreover, in single-digit comparison the reduced model revealed additionally a significant effect of stimulation over right stimulation vs. sham indicating that single-digit number comparison was slower following anodal tDCS over the right IPS as compared to sham.

## Discussion

The aim of this study was to evaluate the causal structure-function relationship between the IPS and basic magnitude processing, separately for each hemisphere. Our confirmatory analyses showed that facilitatory unilateral tDCS over the left or right IPS did not significantly modulate the distance effect. This is at odds with our hypotheses about differential modulation of the distance effect by tDCS. However, in the exploratory analyses when we controlled for the training effect, an effect of stimulation emerged. It revealed that anodal stimulation over the right IPS vs. sham has slowed down single-digit number comparison. We will discuss both outcomes and their theoretical implications in more detail.

Contrary to our expectations, we did not observe a significant modulation of the distance effect by unilateral parietal tDCS over right or left IPS. We hypothesized that the effect of distance may be associated with hemispheric specification with the left IPS subserving small distances^55,56^, and the right IPS large distances^54^. Among other regions in the parietal cortex, activation of the IPS has been associated with the distance effect in the literature^27,28^. However, the fact that the distance effect remained unaffected by unilateral tDCS can be looked at from a different perspective, too: the perspective of effort and automaticity. In our experimental design, the effect of distance was measured by means of participants’ reaction time in a magnitude comparison task in which they had to choose the larger number in a pair. Noticeably, the distance effect is not an effortful sort of magnitude processing. This holds not only for single-digit number comparison but also for the used version of two-digit number comparison because of the absence of within-decade filler trials which allows participants to only focus on the task-relevant decades without processing the whole numbers. Supporting this assumption, the overall mean reaction time was only 610 ms for two-digit numbers compared to the mean reaction time in other numerical comparison tasks with 25% filler trials (800 ms) up to 75% filler trials (900 ms)^73^. As filler trials increase cognitive demands in the magnitude comparison task, our magnitude comparison task without filler trials therefore was an easier version of this task. Thus, our tasks might have been too easy and effortless for excitatory anodal stimulation to improve performance.

Indeed, in more complex numerical tasks such as calculations, the impact of stimulation has been observed. For example, Artemenko et al.^62^ observed a significant effect of anodal vs. cathodal tDCS over the right IPS on the carry effect in a two-digit addition task. The carry effect indicates increased arithmetic difficulty when the sum of the units of the operands exceeds 9. In a similar vein, during an EEG-tDCS study involving anodal left parietal placement, the researchers observed a decreased reaction time in operations with a larger problem size effect (sums exceeding 10 and including the carry operation)^81^. In another subtraction task, the reaction time improved following anodal tDCS over the left frontal area for large number subtraction, but not for small numbers^64^. In sum, in more complex tasks, magnitude-related effects of unilateral stimulation have been found for distance or size.

However, we suggest that when the numerical effect to be modulated is basic or presumably automatic, it is possible that it cannot be more affected by reinforcement, for example brain stimulation. Similar to our non-significant finding, Di Rosa et al.^82^ could not find the effect of anodal or cathodal tDCS on other basic effects such as the SNARC^20^ and the MARC^83^ effects. Yet, Hauser et al.^60^ reported an effect of unilateral tDCS on distance effect—but only in accuracy and not in reaction time. Schroeder et al.^84^ ran a side analysis in a single-digit magnitude comparison while studying the SNARC effect. They only found the SNARC effect but not the distance effect to be manipulated by cathodal tDCS over the left prefrontal cortex. Note that in their study the IPS was not stimulated, only the left prefrontal cortex, and furthermore, cathodal and anodal stimulations are not directly comparable despite their similarities. To summarize, tDCS modulations of simple effects like the distance effect have often not been observed. And if they have been observed for other effects like the SNARC effect, it was not for stimulation of the IPS. Therefore, two-digit number comparison without fillers may have been too automatic and effortless to be influenced by unilateral stimulation of the IPS.

A second consideration, which is crucial to acknowledge that the IPS is not an isolated region in the brain but rather part of a network. Recent functional and structural research extended The Triple Code Model by incorporating the fronto-parietal network of number processing^37,42,85^. Considering the involvement of the fronto-parietal network, it is plausible that the effect caused by unilateral tDCS over the IPS from one hemisphere might not be sufficient to adequately influence magnitude processing. Indeed, some previous tDCS studies^61,62^ corroborating The Triple Code Model^33,43^ suggesting that magnitude information is processed in the IPS bilaterally. Possibly, because of strong reciprocal connections between the left and the right IPS, unilateral stimulation did not modulate the distance effect as expected. What is more, frontal brain regions, associated with domain-general functions including working memory and attention, might be also a target for stimulation, as frontal stimulation effects on numerical cognition have been repeatedly found^84,86^. Consequently, the impact of tDCS on the IPS alone may not be sufficient to modulate magnitude processing effectively, as the broader bilateral fronto-parietal network plays a significant role in this cognitive function. Therefore, future studies might use multichannel stimulation protocols which are able to influence the whole network.

Third, the null effect could be due to the particular stimulation protocol used. We decided to apply a tDCS protocol similar to Hauser et al.^60^, who had found modulation of the distance effect at least for accuracy. Our protocol considered a quite weak current of 1 mA and a low current density of 0.029 mA/cm^2^. Consequently, a possible stimulation effect might not be strong enough—and a stronger tDCS might yield different results. However, this cannot be taken for granted, as tDCS effects on cognition are not linear so that not necessarily the stronger current is more effective^87^. Therefore, for future tDCS studies it might be crucial to apply different currents.

Fourth, repetition and training effects have to be considered. In our study, we used a repeated measures design, in which participants repeated both the single-digit and two-digit magnitude comparison tasks three times. Given the repeated exposure to the task during the three sessions, the cumulative effects of training and task practice were substantial. Thus, the task repetition effect might have masked the stimulation effect. Indeed we found evidence for this idea in an exploratory analysis: After controlling for the effect of training by taking the session as a fixed factor in the LMM, we found a main effect of stimulation. Regardless of distance, single-digit number comparison took longer following the stimulation over the right IPS when compared to sham. This exploratory result indirectly corroborates our hypothesis about the left hemisphere being more involved in small distance and more effortful processing by showing that after excitatory stimulation of the contralateral side, the expected performance has worsened. This result was found specifically for single-digit number processing which can be claimed as less effortful and more automatic compared to two-digit number processing. However, because this analysis was not preregistered and only indirectly corresponds to our hypothesis, we do not want to make a strong claim based on this result. But we believe that this could serve for hypothesis generation for future studies, which might test in a preregistered replication if there is an interaction of hemispheric activation and responses to different number sizes (single-digit vs. two-digit). Finally, it shows that more research is needed to study functional lateralization in numerical cognition.

## Conclusion

In the current study the confirmatory result failed to provide evidence for a hemispheric lateralization of magnitude processing in the IPS. Unilateral tDCS did not modulate the processing of small or large numbers. We offered four possible explanations: (i) The lack of stimulation effects on distance might also be attributed to the relative simplicity of the numerical effect being investigated together with the difficulty of improving performance in healthy, high-functioning subjects. (ii) The IPS is not isolated, but part of a large fronto-parietal network including the contralateral IPS; which could compensate for the lack of an unilateral stimulation effect in the IPS. (iii) Following Hauser et al.^63^, we applied a relatively weak current. Other stimulation parameters, in particular with stronger stimulation might yield different effects. (iv) Finally, the task repetition has masked eventual stimulation effects. Indeed, in an exploratory analysis, we observed a stimulation effect after stimulation of the right hemisphere in single-digit number comparison when controlling for the training effect. However, such an exploratory result does not allow for conclusions on our hypothesis. We conclude that hemispheric specialization for different magnitudes or distance effects cannot be easily shown by unilateral brain stimulation in a relatively effortless task.

We believe that these explanations of the null effect deserve better investigation with additional control experiments studying the exact questions raised above. However, in our view this requires a systematic research program with several studies for each of the four explanations which is unfortunately far beyond the scope of this article. Nevertheless, we are convinced that this research could serve as a starting point for future follow-up experiments. We recommend systematically varying parameters, such as two-digit number comparison tasks with and without filler trials, different stimulation protocols, and various electrode montages, to investigate the effects of task difficulty, current intensity, and the involvement of the entire fronto-parietal network vs. the IPS alone. Furthermore, conducting different analyses (including the consideration of repetitive sessions and training effects) holds promise for investigating the functional lateralization of number processing.

So, while altogether this study raised many new questions, it also contains some clear take-home messages. The distance effect, the hallmark effect of number magnitude processing, which is almost always observed in every experiment cannot be easily differentially altered by lateralized stimulation. This does not preclude that it might be found to be lateralized with subtle variations in stimuli, design and methods, but at least its lateralization is not that strong that it simply prevails largely independent of experimental context.

### Supplementary Information

Supplementary Table 1.

## Data Availability

The datasets are available: (https://osf.io/g57av/).
