# Supplementary material for: tDCS effects in basic symbolic number magnitude processing are not significantly lateralized
Source: Sci Rep. 2023 Dec 6;13:21515. doi: 10.1038/s41598-023-48189-z (PMC10700326; doi:10.1038/s41598-023-48189-z)
Supplement: Supplementary file 1 — Supplementary Table 1. [file 41598_2023_48189_MOESM1_ESM.pdf]

tDCS effects in basic symbolic number magnitude processing are not significantly lateralized

Narjes Bahreini 1,*, Christina Artemenko 1 , Christian Plewnia 2, 3 & Hans-Christoph Nuerk 1, 3 1Department of Psychology, University of Tuebingen, Germany 2Department of Psychiatry and Psychotherapy, Neurophysiology & Interventional Neuropsychiatry, University Hospital of Tuebingen, Germany 3German Centre for Mental Health (DZPG)

Appendix table 1 | Results of the exploratory LMM analysis. Estimates, SE, their respective *t*- and *p*-values of fixed effects, separately for single- and two-digit number comparison.

Task	Effect	Estimate	SE	*df*	*t*-value	*p*
Single-digit	stimulation (left vs. sham)	0.000	0.001	40120	0.708	.479
	stimulation (right vs. sham)	-0.003	0.001	40120	-2.798	< .001
	distance (small vs. large)	0.050	0.000	40120	52.090	< .001
	session (2 vs. 1)	-0.019	0.001	40120	-15.887	< .001
	session (3 vs. 1)	-0.026	0.001	40120	-22.288	< .001
Two-digit	distance (small vs. large)	0.073	0.001	34380	49.294	< .001
	session (2 vs. 1)	0.012	0.001	34380	7.052	< .001
	session (3 vs. 1)	-0.021	0.001	34380	-12.077	< .001
